# Factors Associated with the Initiation of Added Sugar among Low-Income Young Children Participating in the Special Supplemental Nutrition Program for Women, Infants, and Children in the US

**DOI:** 10.3390/nu13113888

**Published:** 2021-10-29

**Authors:** Morium B. Bably, Rajib Paul, Sarah B. Laditka, Elizabeth F. Racine

**Affiliations:** Department of Public Health Sciences, The University of North Carolina at Charlotte, University City Blvd 9201, Charlotte, NC 28223, USA; rpaul9@uncc.edu (R.P.); sladitka@uncc.edu (S.B.L.); efracine@uncc.edu (E.F.R.)

**Keywords:** added sugar, WIC children, initiation of added sugar, US infants and children

## Abstract

Added sugar intake at a young age is associated with chronic diseases including cardiovascular diseases, asthma, elevated blood pressure, and overweight. The Dietary Guidelines for Americans 2020–2025 and the American Heart Association recommend delaying the introduction of added sugar until age 2. The aims of this study were to identify the timing of added sugar initiation; factors associated with added sugar initiation; and the top five added sugar foods and beverages consumed by infants and children at three age ranges (<7 months, 8–13 months, and 14–24 months). Data were from the Special Supplemental Nutrition Program for Women, Infants, and Children (WIC) Infant and Toddler Feeding Practices Study-2, a longitudinal, national population of WIC participants enrolled in WIC eligible clinics (*n* = 3835). The Cox proportional hazards model was used to examine the factors associated with introducing added sugar. About 25% of children were given added sugar at or before 7 months. Contributing factors were caregivers’ race/ethnicity, education, employment, weight status, parity, child sex, and premature birth (all *p* < 0.05). The top added sugar foods consumed between 1–24 months were cereal, crackers, apple sauce, dessert, yogurt, sweetened beverages, syrup and preserves, and cookies. Further research to examine the impact of early initiation of added sugar on health outcomes and taste preferences is warranted.

## 1. Introduction

The United States (US) Dietary Guidelines Advisory Committee and American Heart Association recommend that added sugar intake be limited to <10% of the total energy intake for children 2 years and older [1,2]. This is the equivalent of about 6 teaspoons (25 g) of sugar per day for children aged 2 to 19 years [1,2,3]. The Dietary Guidelines for Americans 2020–2025 recommend avoiding foods and beverages with added sugar for children 2 years and younger [4]. The American Heart Association also suggests parents should not initiate added sugar consumption among children under the age of 2 years [2]. “Added sugar” is often defined as all sugars, syrups, or caloric sweeteners added during food preparation, processing, and food manufacturing [5].

Previous researchers have found that added sugar intake at a young age is negatively associated with health. Higher levels of added sugar intake are associated with an increased risk of dental caries [6], cardio-metabolic risk factors [2], asthma [7], elevated blood pressure [8], excess energy intake, poor diet quality [9], and overweight among children [10]. In addition, there are disparities in added sugar intake by income: lower income children have a higher added sugar intake [10,11]. Higher consumption of added sugar among lower income children contributes to health disparities over the life course [7,8,11].

Research on this topic is sparse. Thus, it is not known how much added sugar children aged 2 years and younger consume and when added sugar was introduced. To our knowledge, there have only been three studies in this area. Herrick and colleagues conducted a cross-sectional study among 1211 children from birth to 23 months old in the US. The researchers found that those aged 12 to 23 months consumed more added sugar per day compared to infants aged birth to <12 months (5.8 vs. 0.9 teaspoons); about 84% of infants and toddlers consumed added sugar on a given day [10]. Au et al. (2018) conducted an observational study to estimate the diet quality among 5955 Special Supplemental Nutrition Program for Women, Infants, and Children (WIC) participant children aged 7 to 24 months. Using the Healthy Eating Index, the authors found that added sugar intake increased as children increased in age from 13 to 24 months [12]. Wang et al. (2018) conducted a cross-sectional study to examine the intake and sources of added sugar, saturated fats, and sodium among non-breastfeeding children aged 5 years and younger in the US. The researchers found that added sugar consumption increased rapidly among infants, from birth to 12 months old, and then more gradually up to the age of 2 years, with the lowest consumption among infants and toddlers born to women with higher income [13]. There is also a lack of research documenting the sources of added sugar foods that children under the age of 2 years consume. Herrick and colleagues (2020) found that the common sources of added sugar intake were yogurt, baby food snacks, and sweet bakery products for infants and fruit drinks and sweet bakery products for toddlers [8]; Wang et al. (2018) identified that the common sources of added sugar among children 5 years and younger were cakes/cookies/pastries/pies, sweets, and fruit juice drinks or fruit-flavored drinks [13].

Previous studies used nationally representative samples of US infants and toddlers to examine the added sugar consumption [8,13]. Although Au et al. (2018) used a sample of WIC participants, their objective was to examine the diet quality of infants and toddlers [13]. No research that we are aware of has examined added sugar initiation and intake among low-income US infants and toddlers. Given the association between added sugar consumption during childhood and related morbidities and dietary habits later in life, it is useful to understand when added sugar consumption begins.

Thus, our study had three objectives: the first was to document the timing of added sugar initiation; the second was to determine individual-level factors associated with added sugar initiation; and the third aim was to identify the top five added sugar foods and beverages (hereafter food items) consumed by low-income infants and children at three age ranges. We used data from the WIC Program, sponsored by the United States Department of Agriculture (USDA). The goal of WIC is to provide nutritious food to supplement diets, information on healthy eating, and referrals to health care for low-income women, infants, and children up to 5 years of age [14].

## 2. Materials and Methods

### 2.1. Data Source and Study Design

The Special Supplemental Nutrition Program for Women, Infants, and Children (WIC) Infant and Toddler Feeding Practices Study-2 (WIC ITFPS-2) is also known as the “Feeding My Baby” study. The WIC ITFPS-2 is a longitudinal national study of caregivers and their children. This study examines feeding practices, associations between WIC services and those practices, and the health and nutrition outcomes of children receiving WIC. The study investigators collaborated with the USDA Food and Nutrition Service, Westat, WIC State Agencies, and WIC sites [14].

During a 20-week window between 1 July 2013 and 18 November 2013, study participants were recruited from 80 large WIC sites across 27 US states and territories. Eligible WIC sites were limited to those expected to enroll at least 30 new study-eligible cases each month. A stratified two-stage sampling methodology was used to choose these WIC sites. Child participants were recruited through interviews with the primary caregiver. To participate in WIC ITFPS-2, women had to be (i) pregnant, or with an infant less than 2.5 months old, (ii) enrolled in WIC for this pregnancy or child for the first time, (iii) at least 16 years old, and (iv) a speaker of English or Spanish. A total of 4367 women were recruited. The children were enrolled while the mother was pregnant, or after birth until the child was aged 2.5 months, and then remained in the study until the child turned 9 years old. Detailed sampling strategies can be found in the WIC ITFPS-2: Infant Year Report [14].

To collect longitudinal data, interviewers conducted follow-up telephone interviews in English or Spanish every 2 to 6 months starting during the prenatal period. Once the child was born, telephone follow-up interviews were conducted when the infants and children were 1, 3, 5, 7, 9, 11, 13, 15, 18, 24, 30, 36, 42, 48, 54, 60, and 72 months of age. The follow-up interviews were scheduled based on the birthday of the children; the interview window started 14 days before the child turns the age of the interview (e.g., 14 days before the child is 24 months old) and closed 28 days after the child turns that age (e.g., 28 days after the child is 24 months old). All caregivers provided written informed consent during enrollment. The data were weighted to represent the national population of caregivers, infants, and children enrolled in WIC-study eligible clinics between July to November 2013, and further adjusted to account for interview nonresponse. The average response rate between enrollment and 24 months was about 60% [14].

All post-birth interviews, except the 30, 42, and 54-month interviews, included a 24-h dietary recall using the Automated Multi-Pass Method (AMPM) to gather information on nutrient intake. The 24-h dietary recall that used the USDA AMPM was administered over phone during the interview. In the AMPM, caregivers were asked to recall and report the child’s dietary intake including all foods, beverages, and dietary supplements for each event during the past 24 h. During the interview, caregivers also received a package of measuring guides to help them report their child’s portion sizes. This information was recorded by the interviewer and then coded and translated into calories, nutrients, and food group value [14].

### 2.2. Sample

Of the total WIC ITFPS-2 participants (*n* = 4367), we excluded those who did not provide information on child added sugar intake from the analysis (*n* = 532). The analytic sample for our study includes information about infants and children from birth to 24 months (*n* = 3835).

### 2.3. Outcome Measures

The outcome measure for objectives 1 and 2 is the month of added sugar initiation. Questions about total daily nutrient intake for 1-month through 24-month-olds were collected via 24-h dietary recall using the USDA’s AMPM. The outcome measure for objective 3 is the top five added sugar food items infants and children consumed. This information was also captured from AMPA where participants reported dietary intake including all foods, beverages, and dietary supplements for each event during the past 24 h.

### 2.4. Age Stratification for Objectives 1 and 3

Based on relevant previous work on infant feeding practices [8,15,16], we categorized infants and toddlers into three age groups: ≤7 months, 8–13 months, and 14–24 months. Infants and toddlers reach different feeding milestones during these three age ranges [17]. From birth to 7 months, infants are introduced to food other than breastmilk (especially between 4–6 months), start learning taste preferences and may easily accept new food, and mostly depend on caregivers for food choice [16]; between ages 8–13 months children start to recognize food by sight, smell, and taste; and between ages 14–24 months children start to develop food preferences and begin rejecting or demanding certain foods, including those with added sugar [17,18,19].

### 2.5. Independent Measures Explored to Address Objective 2

To determine factors associated with the month of added sugar initiation, we examined several individual-level social and demographic factors identified as predictors of diet quality in previous research. Specifically, we studied the following independent measures: age at childbirth (16–19 years, 20–25 years, and 26 years or older) [8]; race/ethnicity (Hispanic, non-Hispanic, non-Hispanic White, non-Hispanic African American, and non-Hispanic others) [10,13]; marital status (married and not married) [10]; education, (high school or less and more than high school) [12]; employment status (full time, part time, and not working) [8,13]; household poverty level (75% of the federal poverty guideline or below, above 75–130% of the federal poverty guideline, above 130% of the federal poverty guideline) [12]; body-mass-index (BMI) status at enrollment (normal or underweight, overweight or obese) [20]; parity (first birth, second birth, third or more birth) [21]; child birth weight (low, normal, and high) [20]; sex of the child (male and female) [8]; and child born before 3 weeks prior due date (yes, no) [22].

### 2.6. Statistical Analysis

To identify the timing of added sugar initiation (objective 1), descriptive analysis was conducted. To determine factors associated with added sugar initiation (objective 2), we used the Cox proportional hazards model (CPHM), adjusted by survey weights. This analysis provided hazard ratios (HRs) and 95% confidence intervals (CIs), where a hazard ratio of more than 1.0 refers to early initiation of added sugar. To determine the variables to keep in the adjusted model, we conducted a bivariate analysis. We kept those that were significant in the bivariate analysis in the adjusted model. We also compared hazard ratios and confidence intervals for the unadjusted and adjusted regression models to assess any meaning changes that could arise due to associations among the independent variables. There were no major statistically significant changes in the hazard ratios, indicating there was no evidence of any meaningful changes. To identify the top five added sugar food items consumed by WIC participants at various age ranges (objective 3), we used descriptive statistics to identify the five food items most frequently consumed at ages ≤ 7 months, 8–13 months, and 14–24 months.

For analyses, statistical significance was determined at *p* < 0.05; all analyses were conducted using weighted procedures that accounted for the complex study design using SAS 9.4. This study was deemed exempt under federal regulations (45 CFR 46.102 (e or l) and 21 CFR 56.102(c)(e)(l)). The Institutional Review Board (IRB) at our university (University blinded) determined that this research did not require IRB review.

## 3. Results

The study sample characteristics are shown in Table 1. Of the 3835 children, added sugar was initiated among 21.7% before or by 7 months, 56.6% between 8 to 13 months, 8.7% between 14 to 24 months of age, and 13% after 24 months of age. About half of the caregivers were 26 years or older (48%) and identified as Hispanic (51%). More than half of the caregivers were not married (67%), not working (61%), had a high school diploma or less (62%), and had income below 75% of the federal poverty level (62%). About half of the children were male (50%) and the majority had a normal birth weight (92%) (Table 1). These characteristics were consistent with those of the participants enrolled in WIC [14].

Of the 11 individual-level factors considered, all were significantly associated with early initiation of added sugar in the adjusted analysis (Table 2). Across racial/ethnic groups, Hispanic, non-Hispanic White, and non-Hispanic others were more likely to introduce added sugar early compared to non-Hispanic African Americans (Hispanic HR 1.30, CI 1.29–1.32; non-Hispanic White HR 1.08, CI 1.07–1.09; non-Hispanic others HR 1.05, CI 1.07–1.09). Caregivers with less than a high school education were more likely to introduce added sugar early compared to caregivers with more education (HR 1.07, CI 1.07–1.08). Caregivers with a household poverty level at <75% of the federal poverty guideline were more likely to initiate early added sugar compared to caregivers with a household poverty level at >130% of the poverty guideline (HR 1.03, CI 1.01–1.04). Caregivers who were overweight or obese were more likely to initiate added sugar (HR 1.04, CI 1.04–1.05). Finally, early added sugar consumption was more likely if a child was born >3 weeks before their due date compared to children who were not born >3 weeks prior to the due date (HR 1.07, CI 1.05–1.08) (Table 2).

The top five added sugar contained food items consumed at 1–7 and 8–13 months were baby cereal, crackers, apple sauce, dessert, and cookies. The top five added sugar contained foods consumed at 14–24 months were crackers, sweetened beverages, yogurt, syrup and preserves, and presweetened cereal (Table 3).

## 4. Discussion

We addressed a notable gap in the literature by exploring the timing of added sugar initiation and the association between social and demographic characteristics and initiation of added sugar intake. The results suggested one-quarter of participants initiated added sugar before or by 7 months. Nearly 90% of children (87%) were given added sugar by 24 months of age.

Individual-level factors significantly associated with added sugar intake at three age ranges among children (race, income, education, child sex) in the present study were consistent with those of previous research [10,13]. In the study by Herrick et al. (2020), the researchers found that non-Hispanic Black women were more likely to consume higher amounts of added sugar compared to people in other racial and ethnic groups [10]. In contrast, we found that non-Hispanic Black women were more likely to delay the initiation of added sugar. Herrick et al. conducted a cross-sectional analysis among infants and toddlers using data from the nationally representative US National Health and Nutrition Examination Survey. The difference in results may be due in part to the fact that our study participants, who are enrolled in WIC, have a notably lower income and less education. Additional research to explore the amount and timing of added sugar intake among different racial and ethnic groups would be useful. We explored several individual-level factors not examined by previous researchers, including parity and if a child was born >3 weeks before their due date.

The top sources of added sugar for infants aged 1–13 months were baby cereal, crackers, apple sauce, dessert, and cookies; top sources for infants and children aged 14–24 months were crackers, sweetened beverages, yogurt, syrup and preserves, and presweetened cereal. Our results for top sources of added sugar were consistent with the study by Herrick et al. (2020), where the participants were of similar ages [10]. However, Wang et al. (2018) identified cakes/cookies/pastries/pies, sweets, and fruit juice drinks or fruit-flavored drinks to be the top sources of added sugar among children 5 years and younger [13]. This finding is useful because this information can help to develop strategic interventions and health promotion campaigns to reduce added sugar intake at a young age. Caregivers may not be aware that the foods they provide contain added sugar. Therefore, it is important to educate caregivers on the sources of added sugar in children’s diets and when these foods are first being introduced into their diet.

Added sugar intake at an early age may have adverse life-long health consequences, including overweight, obesity, cardiovascular diseases, asthma, and dental caries, as well as worse dietary habits [9,10]. With the added sugar consumption, infants and toddlers may not intake the important nutrients that are needed at this age for their overall physical and mental wellbeing [23]. The professional nutrition and health community, specifically the American Heart Association and The US Dietary Guidelines Advisory Committee, recommends that added sugar should not be included in the diet until the age of 2 years; limiting added sugar intake to less than 10%of calories per day starting at the age of 2 years is recommended to reduce the risk of related morbidities [24]. We found that only 13% of WIC children had their added sugar initiation delayed until they were 2 years of age. These findings suggest that lower-income children may be at risk of developing negative health outcomes related to added sugar intake.

Practicing appropriate feeding during infancy is important because children begin to learn food and taste preferences during infancy [18,19]. Eating behaviors developed during this age are more likely to evolve later in life [19]. Thus, caregivers should be aware of the feeding practices since infants are dependent on caregivers for food. Our study results suggest that women with lower-income and less education may be more likely to initiate added sugar into the diets of infants and toddlers. Healthcare professionals and WIC nutritionists can be useful resources to communicate information about the adverse effects of added sugar initiation at an early age. Moreover, intervention is needed to educate the caregivers about the negative health outcomes associated with added sugar consumption at an early age. Previous researchers have proposed effective interventions to improve infant feeding practices, including breastfeeding and bottle-feeding among women participating in WIC [25]. Similar interventions can be adapted to inform the caregivers participating in WIC about the recommendation of added sugar intake and negative health outcomes associated with added sugar intake.

Added sugar intake at a young age causes or exacerbates chronic diseases [2,7,8,10]. The Dietary Guidelines for Americans recognize the importance of evidence-based research and strategies to reduce added sugar intake at a young age. Therefore, evidence-based and theory-based intervention is needed to understand strategies to reduce added sugar intake. Moreover, there remains a lack of research in this area. First, further research is needed to understand the association between added sugar intake during infancy and the preference for added sugar among young children, and if the association has long-term health consequences later in life. Second, qualitative research is needed to understand caregivers’ behaviors associated with introducing added sugar to infants and young children, to understand the degree to which caregivers are aware of the food items that contain added sugar, and to understand if caregivers are aware of the adverse effects of added sugar consumption at young ages. Third, it is important to understand the patterns of added sugar consumption, specifically the variations in volume and frequency of added sugar intake and to examine whether different added sugar intake patterns influence growth and health outcomes. Fourth, future research to better understand why women in some sub-groups initiate early added sugar more than others would be useful. Fifth, it would be useful for future research to examine if the COVID-19 pandemic was associated with changes in added sugar consumption among children. Lastly, it is noteworthy to mention that when the WIC ITFPS-2 interviews began in July 2013, there were no formal recommendations about added sugar intake for infants and children from birth to 2 years old. The American Heart Association provided its recommendations in 2017 [2] and the Dietary Guidelines for Americans provided its recommendations in December 2020 [4]. Thus, it would be useful to extend this research in the future to understand the timing of added sugar initiation after these recommendations were made [25,26].

### Strengths and Limitations

Our study has several strengths. To our knowledge, this is the first study to examine the initiation of added sugar intake and to identify the factors associated with the initiation of added sugar intake among a national population of WIC participants. The study findings are generalizable to low-income populations in the US enrolled in WIC. Our study is also the first to examine three age ranges of infants and toddlers. We also explored several other factors that have not been considered in the existing related research that were found as potential risk factors for the initiation of added sugar at an early age.

Our study also has limitations. The information about added sugar was collected by the 24-h recall method. While this method is widely used to identify food, energy, and nutrient intake it also has limitations. The 24-h recall method depends on participants’ memory; participants may not accurately recall food consumption. Moreover, since the information about added sugar initiation is self-reported, response bias is possible. Finally, the study was conducted among the WIC population. Thus, the study findings are not generalizable to the US population of children.

## 5. Conclusions

In conclusion, the results of our study suggest that added sugar initiation begins during infancy among lower income children. The contributing factors associated with added sugar intake at three age ranges among infants and young children include race/ethnicity, income, education, parity, and child sex. Considering the adverse effect of added sugar, it would be useful to better understand the added sugar intake behavior and added sugar feeding behavior among children and caregivers and how this behavior may influence children’s diet quality and their development of eating behavior later in life. The study results indicate a need for health care advocacy programs and intervention to educate caregivers to stop or limit added sugar consumption among infants and children aged 2 or younger.

## Figures and Tables

**Table 1:** Social and demographic characteristics for participants in WIC ^1^ Infant and Toddler Feeding Practices Study-2, by month of added sugar initiation, *n* = 3835.

	Study Participants Weighted Column % *n* = 3835	Earliest Reported Feeding of Added Sugar to Infant/Toddler ≤ 24 Months ^2^ Weighted Column % *n* = 3328 (87%)			Introduced Added Sugar after 24 Months ^2^ *n* = 507 (13%)
Characteristics ^3^	Birth to 24 Months	≤7 Months n (%) 834 (25)	8 to 13 Months n (%) 2155 (65%)	14 to ≤24 Months n (%) 339 (10)	>24 Months
Age of caregiver at childbirth					
16–19 years	450 (12.8)	120 (15.7)	221 (9.7)	43 (13.0)	76 (18.0)
20–25 years	1591 (39.5)	355 (40.7)	887 (39.1)	144 (39.3)	205 (42.6)
26 years or older	1779 (47.6)	358 (43.5)	1057 (51.2)	152 (47.7)	212 (39.4)
Caregiver’s race/ethnicity					
Hispanic	1560 (50.7)	327 (51.0)	871 (49.0)	147 (53.0)	215 (50.0)
Non-Hispanic White	1018 (23.9)	230 (23.0)	586 (26.1)	59 (15.8)	143 (27.0)
Non-Hispanic African American	885 (18.1)	198 (19.3)	502 (18.2)	96 (22.1)	89 (15.4)
Non-Hispanic others	326 (7.2)	70 (6.8)	174 (6.7)	30 (9.1)	52 (7.6)
Marital status					
Married	1148 (33.2)	225 (30.6)	687 (34.1)	94 (38.7)	142 (27.3)
Not married	2672 (66.8)	608 (69.4)	1468 (65.9)	245 (61.3)	351 (72.7)
Caregiver’s education					
High school or less	2368 (61.5)	533 (68.4)	1279 (58.3)	228 (68.5)	328 (73.5)
More than high school	1442 (38.5)	298 (31.6)	872 (47.7)	110 (31.5)	162 (26.5)
Caregiver’s employment status ^4^					
Full time (35 h or more)	669 (19.8)	151 (17.4)	412 (19.2)	61 (25.5)	45 (24.7)
Part time	625 (18.9)	168 (18.9)	375 (19.4)	52 (12.3)	30 (19.5)
Not working	1829 (61.3)	480 (63.8)	1123 (61.4)	143 (61.9)	83 (53.8)
Household poverty level at enrollment					
<75% of poverty guideline	2415 (61.8)	563 (68.8)	1318 (60.5)	219 (59.3)	315 (62.1)
75–130% of poverty guideline	1035 (27.3)	207 (22.9)	594 (27.8)	99 (32.9)	135 (26.6)
>130% of poverty guideline	385 (10.9)	64 (8.4)	243 (11.7)	21 (7.8)	57 (11.2)
Mother’s Body Mass Index category at screening					
Normal or underweight	1716 (46.1)	578 (29.7)	1197 (55.5)	155 (46.2)	253 (48.3)
Overweight or obese	2119 (53.9)	683 (70.3)	958 (44.5)	205 (53.8)	254 (50.1)
Parity					
First birth	1593 (43.0)	375 (44.9)	891 (42.5)	123 (35.0)	204 (41.9)
Second birth	1030 (26.1)	206 (26.3)	584 (25.8)	108 (35.0)	132 (25.4)
Third or more birth	1197 (30.9)	252 (29.8)	680 (31.7)	108 (30.0)	157 (32.7)
Childbirth weight					
Low	278 (6.8)	57 (7.4)	153 (7.3)	30 (8.9)	38 (7.1)
Normal	3492 (92.1)	766 (91.4)	1972 (91.6)	306 (90.3)	448 (92.1)
High	50 (1.1)	10 (1.2)	30 (1.1)	3 (0.88)	7 (0.77)
Child sex					
Male	1961 (50.3)	428 (48.8)	1089 (49.2)	184 (57.9)	260 (46.9)
Female	1874 (49.7)	406 (51.3)	1066 (50.8)	155 (42.1)	247 (48.7)
Baby born >3 weeks before due date					
Yes	356 (9.2)	59 (9.6)	218 (10.2)	41 (14.4)	38 (8.0)
No	3068 (90.7)	690 (90.4)	1738 (89.8)	258 (85.6)	382 (92.0)

^1^ Special Supplemental Nutrition Program for Women, Infants, and Children; ^2^ the interview could be administered 14 days before and 28 days after the child turns the age of the interview; ^3^ missing observations range from 0% to 10% depending on the variable, unless otherwise noted; ^4^ missing 714 responses, 18.6% of the sample.

**Table 2:** Adjusted ^1^ Cox proportional hazards model showing the associations between initiation of added sugar and the participant characteristics, WIC ^2^ Infant and Toddler Feeding Practices Study-2.

Characteristics	Hazards Ratio	(95% CI)	*p*-Value
Age of caregiver at childbirth			
16–19 years	0.90	0.89–0.91	<0.0001
20–25 years	0.94	0.93–0.95	<0.0001
26 years or older	Reference	Reference	
Caregiver’s race/ethnicity			
Hispanic	1.30	1.29–1.32	<0.0001
Non-Hispanic White	1.08	1.07–1.09	<0.0001
Non-Hispanic African American	Reference	Reference	
Non-Hispanic others	1.05	1.04–1.07	<0.0001
Marital status			
Married	Reference	Reference	
Not married	1.01	1.00–1.02	0.0361
Caregiver’s education			
High school or less	1.07	1.07–1.08	<0.0001
More than high school	Reference	Reference	
Caregiver’s employment status			
Full time (35 h or more)	1.13	1.12–1.14	<0.0001
Part time	1.11	1.10–1.12	<0.0001
Not working	Reference	Reference	
Household poverty level at enrollment			
<75% of poverty guideline	1.03	1.01–1.04	<0.0001
75–130% of poverty guideline	1.00	0.99–1.01	0.4614
>130% of poverty guideline	Reference	Reference	
Caregiver’s weight status			
Normal or underweight	Reference	Reference	
Overweight or obese	1.04	1.04–1.05	<0.0001
Parity			
First birth	Reference	Reference	
Second birth	0.95	0.94–0.96	<0.0001
Third or more birth	0.94	0.93–0.95	<0.0001
Child birthweight			
Low	0.90	0.89–0.92	<0.0001
Normal	Reference	Reference	
High	0.97	0.94–1.00	0.0232
Child sex			
Male	Reference	Reference	
Female	1.04	1.04–1.05	<0.0001
Premature birth			
Yes	1.07	1.05–1.08	<0.0001
No	Reference	Reference	

^1^ Adjusted for caregiver’s age at childbirth, race/ethnicity, marital status, education, employment status, household poverty level, weight status, parity, childbirth weight, child sex, and premature birth; ^2^ Special Supplemental Nutrition Program for Women, Infants, and Children.

**Table 3:** Top added sugar foods consumed by age (months). WIC ^1^ Infant and Toddler Feeding Practices Study-2.

Ranking	From Birth–7 Months Fed Any Added Sugar *n* = 834		Top Added Sugar Food Intake between 8–13 Months *n* = 2989		Top Added Sugar Food Intake between 14–24 Months *n* = 3328	
	Food Item	% of Children Fed This Item	Food Item	% of Children Fed This Item	Food Item	% of Children Fed This Item
1	Baby cereal	64.9	Baby cereal	58.8	Crackers	47.0
2	Crackers	42.1	Crackers	50.0	Sweetened beverage	27.0
3	Apple sauce	30.6	Apple sauce	31.0	Yogurt	25.4
4	Dessert	26.6	Dessert	30.0	Sugar, syrup, preserves	23.8
5	Cookie	19.8	Cookie	22.2	Presweetened cereal	23.0

^1^ Special Supplemental Nutrition Program for Women, Infants, and Children. Note: Children may have been fed multiple added sugar foods, e.g., a child between the ages of 14–24 months may have been fed crackers and yogurt at some point between 14–24 months.

## Data Availability

The WIC ITFPS-2 data are available upon request from the United States Department of Agriculture.
